# On the Use of Enemata; Particularly in Cases of Gastritis, Enteritis, &c.

**Published:** 1800-03

**Authors:** S. C. Blyth

**Affiliations:** Yarmouth


### *To the Editors of the Medical and Phyſical Journal.*

GENTLEMEN,

I Take the liberty to ſend you a few obſervations for inſertion in your uſeful Work. I cannot recommend them to your notice on the ſcore of novelty; and whether they merit attention on any other claim, I ſubmit to your better judgment. I am,

GENTLEMEN,

With much conſideration,

Your very humble ſervant,

*Yarmouth, Jan.* 1800. S. C. BLYTH.

---

### *On the Uſe of Enemata; particularly in Caſes of Gaſtritis, Enteritis, &c.*

It is a matter of ſurprize to foreigners, that a mode of adminiſtering medicines ſo ancient, and withal ſo common in other other countries, ſhould be comparatively rare in England. While people of other nations make uſe of Enemata as a domeſtic luxury, often independently of any real ailment; many practitioners in this country frequently ſuffer a patient who can keep nothing on his ſtomach, to languiſh in agonizing ſuſpenſe, before they will order for his relief, a remedy of the moſt obvious and ſalutary kind.

No mode of exhibiting medicines can be more comprehenſive than clyſters; they embrace almoſt every article of the Materia Medica, as well as of aliment, whoſe virtues can be imparted to a liquid. Beſides this, no great nicety is neceſſary in this preparation; which is an eſſential advantage when one conſiders the adulteration, and the change or loſs of virtue, which medicines of importance too frequently undergo, in order to be accommodated to a ſqueamiſh palate, or what is ſtill worſe, to a capricious eye.

But the great advantage of this exhibition appears in caſes (and thoſe happening every day), wherein, from the exceſſive irritability of the ſtomach, nothing can be retained on it. This is not only the caſe in diſeaſes which have brought the patient very low, but in many others in an incipient ſtate; and this from a native irritability of fibre. Nor is this a matter new or extraordinary, as its truth every practitioner of experience can atteſt.

In inflammations of the ſtomach and bowels, I have been aſtoniſhed at the ſudden and happy changes effected by clyſters.

Among other caſes, an elderly gentleman in my neighbourhood was ſeized with the ordinary ſymptoms of Gaſtritis,—nauſea, difficulty of breathing, a ſenſe of intenſe heat about the præcordia, thirſt, and coldneſs of the extremities. I treated him according to the antiphlogiſtic plan in general; ordered pediluvia, warm fomentations, &c. but without relief. I then exhibited a laxative and antiſpaſmodic Enema; the ſymptoms immediately abated in their ſeverity, and he gradually recovered.

There are, indeed, few caſes in which clyſters of ſome ſort are contra-indicated. The eminent ſuperiority they claim is chiefly on the ſcore of their innocent operation. In caſes where they do no good, they ſeldom do harm, unleſs their compoſition be abſolutely poiſonous. In cholera morbus, when the patient has been nearly exhauſted, when hiccough and convulſions have beſpoke the deſperate ſtate of his caſe, timely exhibitions of this ſort have ſaved his life. In obſtinate colics too, clyſters of this deſcription have effected a ſalutary evacuation, after all other means had failed. In affections of the bladder, and in

ſuppreſſions

ſuppreſſions of urine from ſpaſm, Enemata have been of ſurpriſing efficacy. In many fevers, ſometimes in typhus, when tonics could be adminiſtered no other way, they have in this ſhape anſwered the beſt of purpoſes.

In the obſtinate intermittents of Carolina and Georgia, how frequently caſes have occurred when the patient was ſo exceſſively debilitated, and his ſtomach had ſo loſt its tone, that no medicine of the corroborant kind would ſtay a moment upon it; and when he has been expecting a renewed attack to carry him off, how often has a ſtrong decoction of the bark, exhibited in this form, preſerved him from the threatened fit, and ſecured his perfect recovery!

In the bilious and yellow fevers of the Weſt Indies, wherein the ſymptoms of approaching death ſucceed each other with melancholy rapidity, the happieſt conſequences have followed theſe exhibitions; and it is unqueſtionable, that the ſucceſs of medical practitioners in the French iſlands, in treating the diſorders of hot climates, may be principally aſcribed to this mode of adminiſtering medicines. From the moſt inconſiderable head-ach to a ſanguineous apoplexy, the French preſcribe Enemata. Machines for this purpoſe are kept in every family, and are viewed with the familiarity of a culinary utenſil. So common a remedy indeed is it, that if one complain, either in France or the colonies, of the ſlighteſt indiſpoſition, *un lavement* is ſure to be recommended. They conceive that moſt complaints originate in ſome inteſtinal obſtruction, (as, in fact, they often do) and that clyſters are a natural cleanſer, as the word imports. Viewed as a mode of giving food, caſes abound, in which patients have been kept alive for weeks by nouriſhing clyſters, when aliment could be adminiſtered in no other way.

It is unneceſſary here to arrange the various formulæ of Enemata in vogue; they are not confined to number nor quality; they comprehend (to repeat what has been before aſſerted) almoſt every article of an alimentary and medicinal nature.

